# Acute basophilic leukemia associated with the t(16;21)(p11;q22)/*FUS*‐*ERG* fusion gene

**DOI:** 10.1002/ccr3.1219

**Published:** 2017-10-11

**Authors:** Yusuke Toda, Yuya Nagai, Daiki Shimomura, Chiyuki Kishimori, Katsuyo Tsuda, Katsuhiro Fukutsuka, Masahiko Hayashida, Hitoshi Ohno

**Affiliations:** ^1^ Department of Hematology Tenri Hospital Tenri Nara Japan; ^2^ Department of Laboratory Medicine Tenri Hospital Tenri Nara Japan; ^3^ Tenri Institute of Medical Research Tenri Nara Japan

**Keywords:** Acute basophilic leukemia, electron microscopy, metachromasia, t(16;21)(p11;q22) translocation, the *FUS*‐*ERG* fusion gene

## Abstract

We herein report a rare case of acute basophilic leukemia with t(16;21)(p11;q22) generating the *FUS*‐*ERG* fusion gene. The basophilic nature of leukemia blasts was demonstrated by cytomorphology, toluidine blue metachromasia, mature basophil‐associated antigen expression, and characteristic granules under electron microscopy. The molecular link between t(16;21)/*FUS*‐*ERG* and basophilic differentiation remains unclear.

## Introduction

Acute basophilic leukemia (ABL) is listed as a separate entity in the acute myeloid leukemia (AML), not otherwise specified category in the 2008 WHO classification, comprising <1% of all cases of AML 1. The very recently proposed criteria of ABL are blasts ≥20% and immature basophils ≥40% of nucleated bone marrow (BM) or peripheral blood (PB) cells 2. However, as the disease is defined on the basis of morphology, limited information is currently available on specific cytogenetic and molecular markers.

The t(16;21)(p11;q22) translocation was initially described in a patient with AML characterized by a large number of abnormal eosinophils in BM 3. Sixty‐six cases carrying t(16;21) are currently listed in the Mitelman database, which shows that this translocation is present in all ages and is associated with poor survival 4. In 1994, two independent groups found that t(16;21) led to the generation of a fusion gene between *FUS* (FUS RNA binding protein) at 16p11 and *ERG* (ERG, ETS transcription factor) at 21q22, encoding the FUS‐ERG chimeric protein 5, 6. The leukemogenic activity of this chimeric protein was demonstrated by the retroviral transduction of *FUS*‐*ERG* to human umbilical cord blood cells, showing altered myeloid and arrested erythroid differentiation and marked increases in the proliferative and self‐renewal capacities of transduced myeloid progenitors 7.

We herein report a rare case of ABL carrying the t(16;21)(p11;q22)/*FUS*‐*ERG* fusion gene. Cytomorphological, electron microscopic, and cytogenetic features as well as the treatment course are described.

## Case Report

A 65‐year‐old man was admitted to the Hematology Department of our hospital with a diagnosis of acute leukemia. Two weeks before, he developed swelling in the right lower leg that extended to the ankle and foot. He presented to the Orthopedic Department, and a blood examination revealed marked leukocytosis. There was no surface lymphadenopathy or splenomegaly. He did not have any symptoms suggestive of the excess release of histamine.

His hemoglobin level was 10.1 g/dL, white cell count 73.77 × 10^9^/L, containing 91.2% blasts, and platelet count 31 × 10^9^/L. His lactate dehydrogenase level was 1850 U/L, C‐reactive protein level 1.7 mg/dL, and fibrin degradation products 6.6 *μ*g/mL. The mRNA level of the Wilms’ tumor‐1 gene was 2.8 × 10^5^ copies/*μ*g RNA.

**Figure 1:**
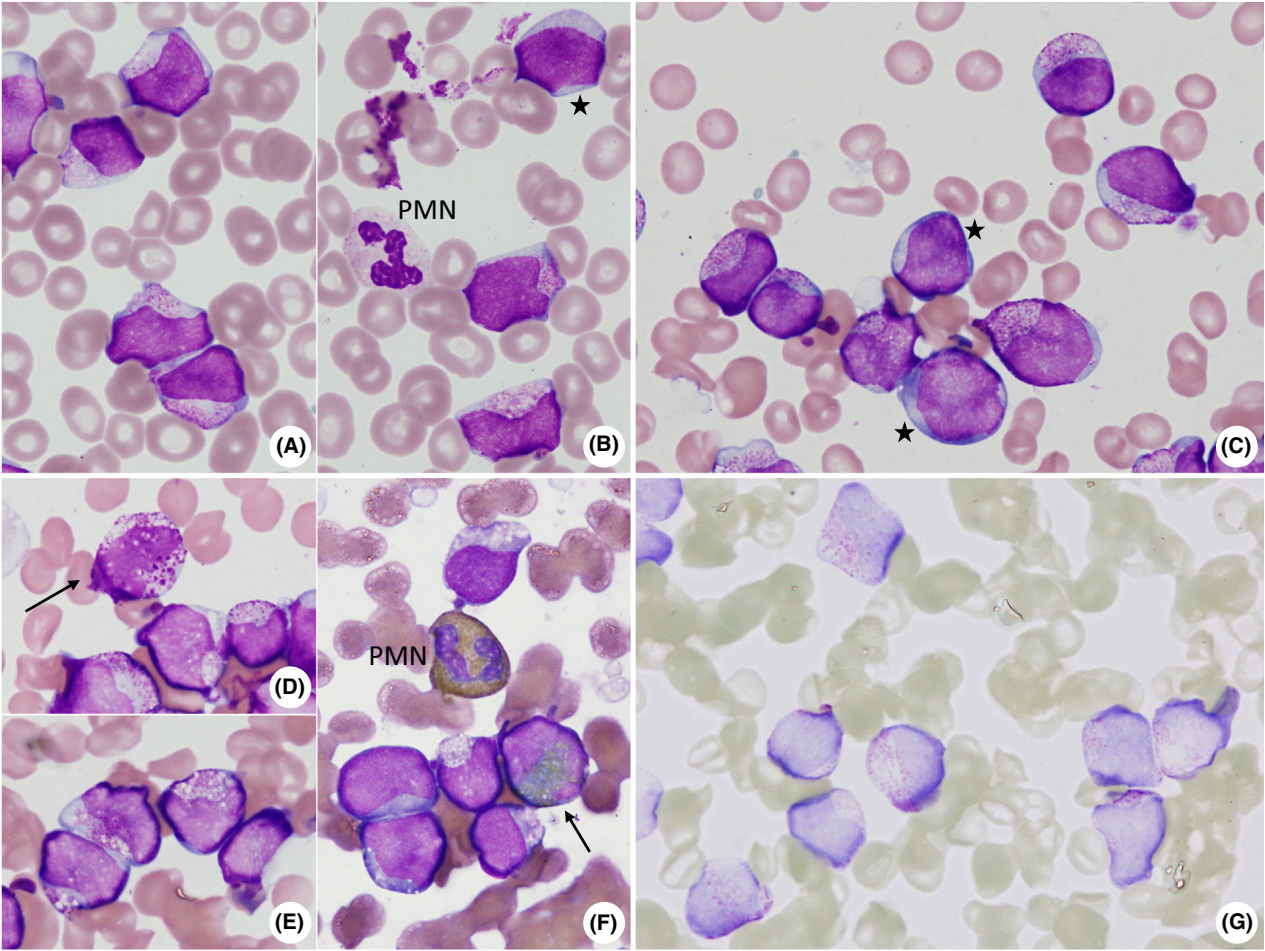
Appearance of leukemia blasts in PB and BM. (A and B) Wright‐stained leukemia blasts in PB with variable amounts of basophilic granules in the cytoplasm, an agranular blast (asterisk), and polymorphonuclear neutrophil (PMN). (C to E) Wright‐stained leukemia blasts in BM, ranging from agranular blasts (asterisks in C) to mature basophils (arrow in D). The granular content appears to be extracted to leave vacuoles in the cytoplasm (E). A fraction of leukemia blasts shows positivity for MPO (arrow in F), albeit weaker than that in PMN. The granules exhibit metachromasia, that is stained magenta, for toluidine blue staining (G). Original magnification ×100 objective lens.

Bone marrow showed 30~40% cellularity containing 95.0% blasts. Leukemia blasts in PB and BM were of medium size and had round or indented nuclei with dispersed chromatin. Nucleoli were inconspicuous. Approximately, 60% of PB and 70% of BM blasts had variable amounts of a light‐blue cytoplasm containing coarse basophilic granules (Fig. 1A–C), and occasional cells showed a mature basophil appearance (Fig. 1D). The granular content often appeared to be extracted to generate cytoplasmic vacuoles (Fig. 1E). No myelodysplastic or hemophagocytic pictures were found. Although 31% PB and 9% BM blasts were positive for myeloperoxidase (MPO), positivity was weak (Fig. 1F). Cytoplasmic granules exhibited metachromasia with toluidine blue staining in 49% PB and 47% BM blasts (Fig. 1G). Chloroacetate esterase and a naphthyl butylate esterase were negative.

## Differential Diagnosis and Investigations

**Figure 2:**
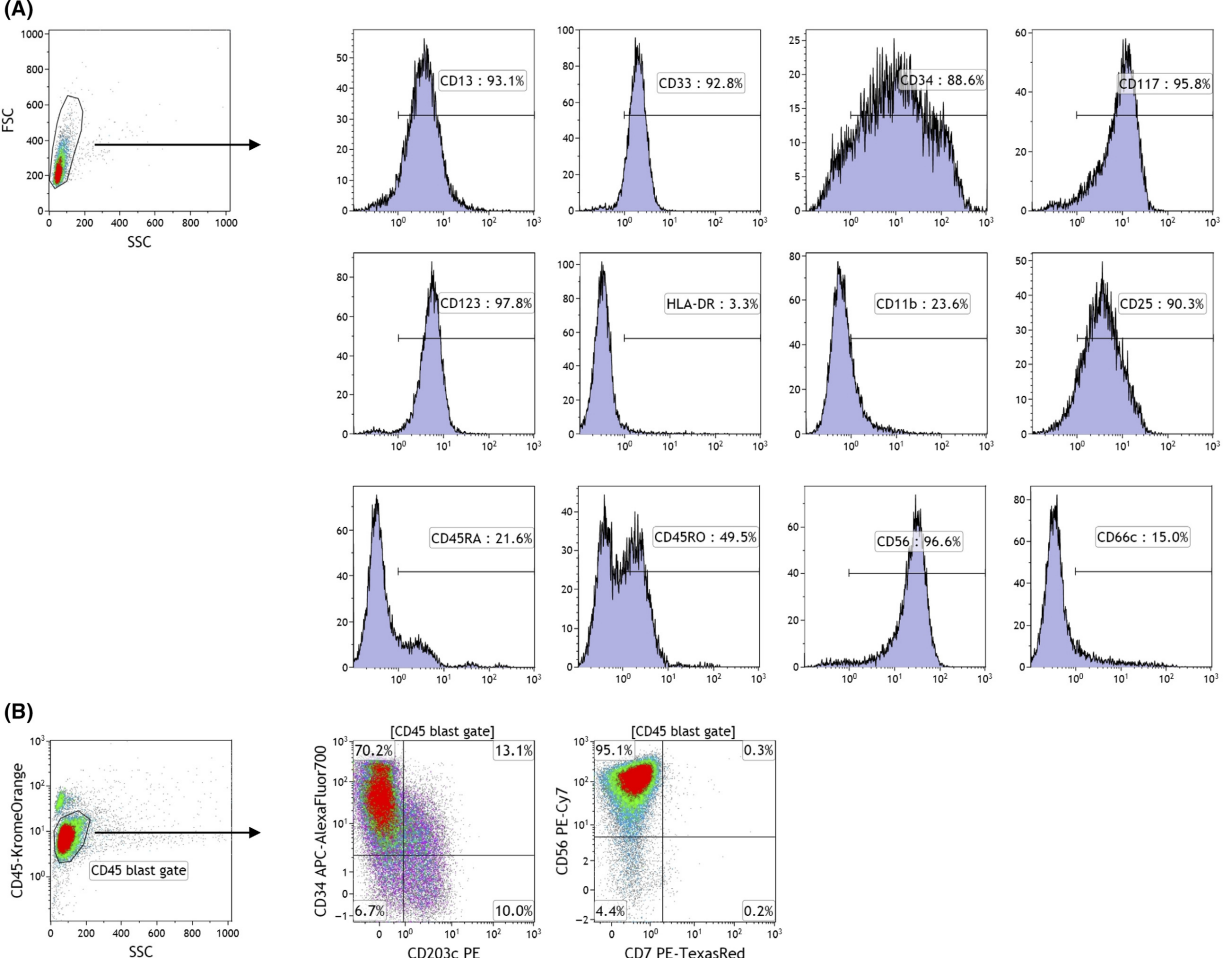
Surface antigen expression of leukemia blasts in BM. (A) Single‐color FCM of leukemia blasts gated by SSC/FSC characteristics. Positive cell populations for each antigen are indicated by horizontal bars, and their percentages are shown. (B) Multicolor FCM of leukemia blasts gated by the weak expression of CD45 and low SSC characteristics. A CD203c/CD34 dot plot shows a broad range of expression levels of both antigens, while a CD7/CD56 dot plot shows the homogeneous expression of CD56 and lack of CD7. The percentages of each quadrant are shown.

Flow cytometry (FCM) revealed that leukemia blasts showed an immature myeloid cell phenotype, that is CD13^+^, CD33^+^, CD34^+^, and CD117^+^, but lacked HLA‐DR expression (Fig. 2A). Approximately, 20% of cells expressed CD203c, which was previously reported to be specific for basophils in PB and increased in response to IgE‐dependent cell activation 2, and the acquisition of the antigen appeared to correlate with the loss of CD34 (Fig. 2B). Other antigens expressed were CD7^−^, CD11b^dim^, CD25^+^, CD45RA^dim^, CD45RO^+^, CD56^+^, CD66c^−^, and CD123^+^.

**Figure 3:**
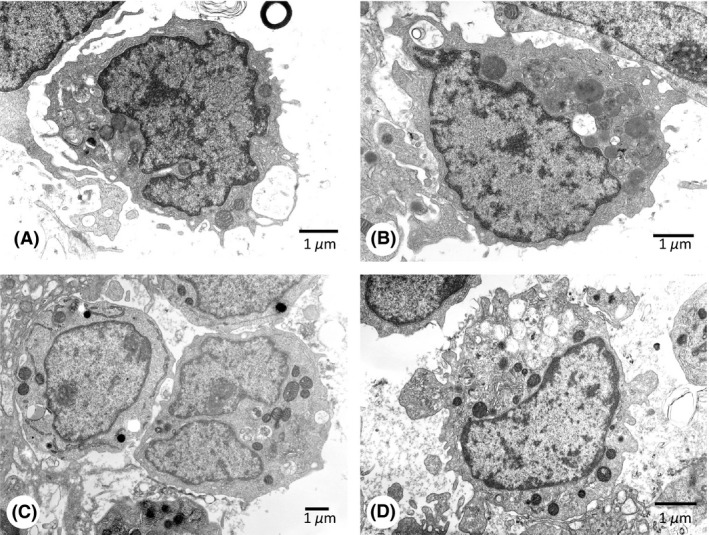
Electron microscopic appearance of leukemia blasts in BM. (A and B) Immature basophils containing cytoplasmic granules of 300–700 nm in diameter. The contents of the granules show a variable appearance, that is those with a similar degree of electron density to that of the cytoplasm, with more intense electron density, a speckled appearance, or electron‐lucent appearance resulting from the extraction of the materials. Occasional granules show myelin‐like figures. Uranyl acetate‐lead citrate staining. (C) MPO reaction in a myeloblast (*left*) and immature basophil (*right*). The myeloblast contains MPO‐positive primary granules in the cytoplasm and shows positivity in the nuclear cistern and rough ER, while the immature basophil was negative for the reaction. (D) An MPO‐stained immature basophil containing abundant granules with a variable appearance. The contents of the granules are negative for the MPO reaction, in contrast to an earlier study.

Electron microscopy showed that leukemia blasts in BM were 6–12 *μ*m in diameter, and the nuclear‐cytoplasmic ratio ranged between 0.5 and 0.9. Cells had round, indented, or irregular nuclei with slightly condensed chromatin and small to medium‐sized nucleoli. In the cytoplasm, variable amounts of mitochondria, rough endoplasmic reticulum (ER), and Golgi apparatus were found among the cells. Twenty to 30% of leukemia blasts contained granules that were 0.3–1.2 mm in diameter with a variable appearance, representing immature basophils (Fig. 3A and B) 8, 9, 10. The materials in the granules showed a similar electron density to the cytoplasm, more intense electron density, or a speckled appearance, and the materials were often extracted to display an electron‐lucent appearance. Occasional granules contained myelin‐like structures. Theta granules were not apparent. On the other hand, approximately 40% of the cells had primary granules of 0.25–0.6 mm in diameter, and MPO activity in these cells was detected in the nuclear cistern, rough ER, Golgi apparatus, and/or primary granules, showing the features of myeloblasts (Fig. 3C). In contrast, the contents of the granules as well as cytoplasmic organelles in immature basophils lacked MPO activity (Fig. 3C and D).

**Figure 4:**
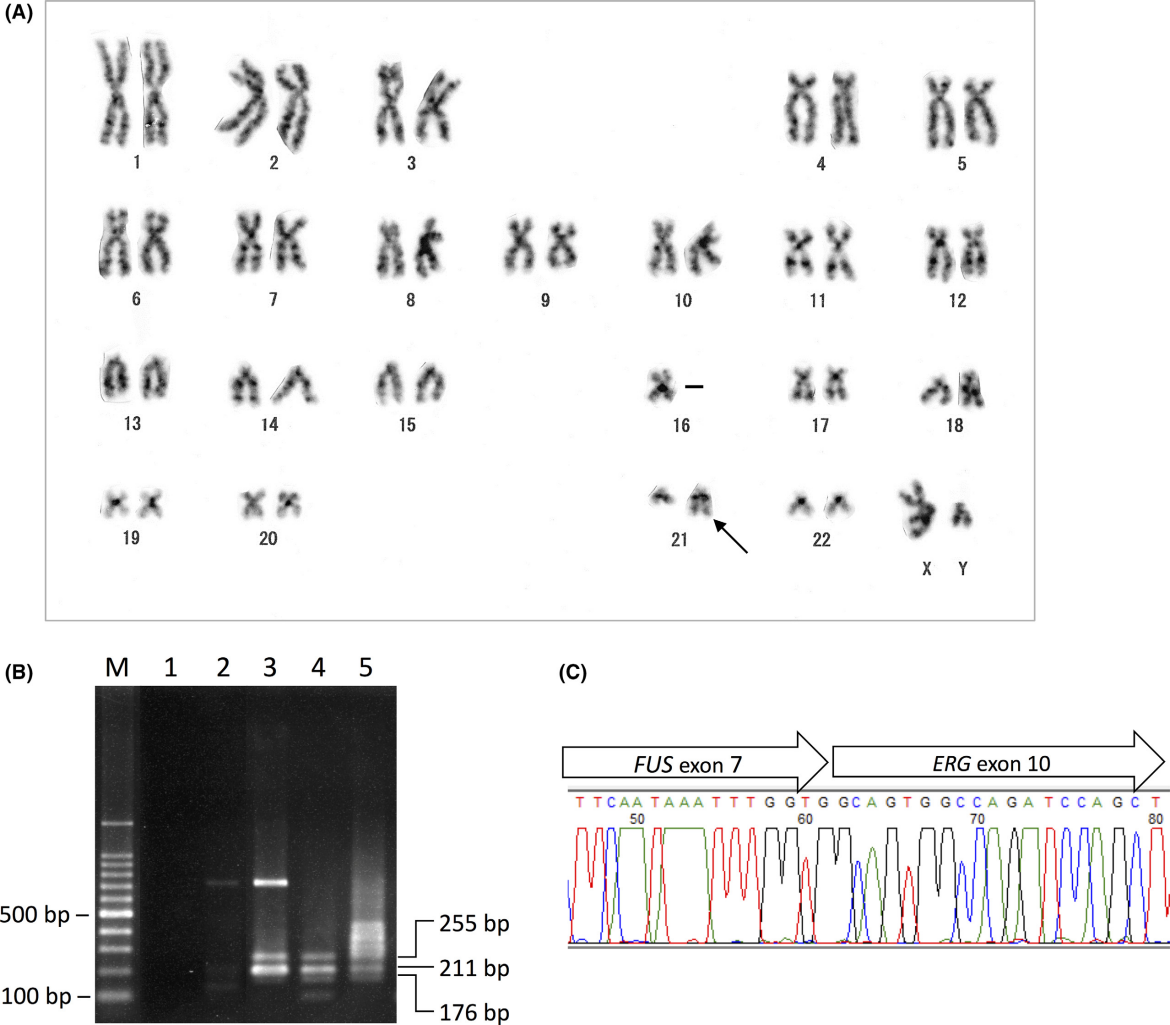
Cytogenetic analysis and RT‐PCR of the *FUS*‐*ERG* fusion gene. (A) G‐banding karyotype obtained from the bone marrow at initial presentation. The arrow indicates the derivative chromosome 21 resulting from the translocation of the chromosome 16 segment distal to band p11 to the long arm of chromosome 21 at band q22 [der(21)t(16;21)(p11;q22)], while the reciprocal der(16)t(16;21)(p11;q22) is deleted. (B) RT‐PCR of the *FUS*‐*ERG* fusion mRNA. Lane M, A 100‐bp ladder molecular weight marker; lane 1, distilled water; lane 2, chronic myeloid leukemia cells as a negative control; lane 3, this case at presentation; lane 4, ×10 dilution of the cDNA material of lane 3; and lane 5, after the idarubicin–cytarabine induction treatment. (C) Nucleotide sequencing encompassing the *FUS*‐*ERG* junction.

G‐banding of the metaphase spreads obtained from the BM specimen revealed the der(21)t(16;21)(p11;q22) chromosome, while the reciprocal der(16)t(16;21)(p11;q22) was deleted. The karyotype according to the ISCN (2013) 11 was 45,XY,−16,der(21)t(16;21)(p11;q22)[12] (Fig. 4A). A reverse transcriptase‐mediated polymerase chain reaction (RT‐PCR) using the nested primer pairs for the *FUS*‐*ERG* fusion transcripts generated three species of DNA, with molecular sizes of 255, 211, and 176 bp, as described earlier (Fig. 4B) 12. The products were subjected to direct sequencing, demonstrating the in‐frame junction encompassing *FUS* exon 7 and *ERG* exon 10 (Fig. 4C).

## Treatment and Outcomes

Although the initial treatment regimen consisting of idarubicin and cytarabine failed to eradicate leukemia cells, MEC (mitoxantrone, etoposide, and cytarabine) salvage treatment led to a hematological response. Cellulitis of the leg resolved during the induction treatment. After three cycles of MEC, *FUS*‐*ERG* fusion mRNA fell to below the level of detection. The patient then underwent hematopoietic stem cell transplantation from his daughter, who was HLA‐A locus mismatched in the graft‐versus‐host direction and HLA‐C and DR loci‐mismatched in the host‐versus‐graft direction. Although the course of the transplant was uneventful, his leukemia briefly relapsed on day 56 of transplantation. A reduction in the dosage and withdrawal of tacrolimus led to the elimination of circulating leukemia blasts; however, he developed severe graft‐versus‐host disease involving the entire gastrointestinal tract. He finally died of *Pseudomonas aeruginosa* septicemia on day 419 of the initial presentation.

## Discussion

We herein described a patient with a rare type of AML characterized by the marked basophilic differentiation of leukemia blasts and the t(16;21)(p11;q22)/*FUS*‐*ERG* fusion gene. Basophilic features were clearly demonstrated by (1) cytomorphological differentiation from agranular blasts to mature basophils, (2) toluidine blue metachromasia, (3) the expression of mature basophil‐associated antigens, that is CD123 and CD203c, and (4) the presence of characteristic granules under electron microscopy.

Chromosomal translocations in AML with the proliferation of basophils are heterogeneous. t(X;6)(p11;q23) is recurrently observed in male infants with ABL, and this translocation generates the fusion gene between *MYB* at 6q23 and *GATA1* at Xp11 13, 14, thereby inducing interleukin receptor‐like 1 and neurotropic receptor tyrosine kinase 1, both of which have been implicated in basophilic differentiation 15. The presence of Philadelphia chromosome, t(9;22)(q34;q11), in some cases of ABL suggests that these cases represented the de novo occurring accelerated/blastic phase of chronic myeloid leukemia (CML) with the preferential proliferation of mature and immature basophils 16. Recent findings suggest that *IKAROS* alterations are associated with disease acceleration and basophil expansion in CML 17. Basophilic differentiation may occur in AML carrying t(8;21)(q22;q22)/*RUNX1*‐*RUNX1T1* 18, 19; however, reactive BM basophilia in response to a factor produced by leukemia blasts was suggested in one case 19. t(6;9)(p22;q34)/*DEK*‐*NUP214* is observed in AML with or without monocytic features and 44–62% of cases show >2% basophilia in PB and BM 20. Other sporadically reported translocations in ABL include t(7;8)(q32;q13), t(2;6)(q23?4;p22?3), and t(6;12)(q13;p13.3) resulting in the loss of *ETV6* 9, 21, 22.

t(16;21)(p11;q22) has been identified in AML with any French American British (FAB) subtype of AML other than FAB‐M3, and the disease may be preceded by myelodysplastic syndrome. In rare cases, this translocation has been observed in acute lymphoblastic leukemia, acute megakaryoblastic leukemia, and the blast crisis of CML 4, 12, 23, 24, 25. The cytomorphology of AML with t(16;21) has been characterized by monocytoid features, the erythrophagocytosis of leukemia blasts, and increased eosinophils with sometimes abnormal features in BM 3, 4, 26, 27. In a single case, increased basophils were observed in PB and BM 23; however, it was not clearly stated whether the case represented AML with basophilic differentiation or if basophilia was reactive. In the present case, leukemia blasts with t(16;21) showed marked basophilic differentiation, matching the diagnostic criteria of ABL 2. Nevertheless, as basophilic differentiation has not been described in *FUS*‐*ERG*–transduced human cord blood cells 7, the molecular link between t(16;21)(p11;q22)/*FUS*‐*ERG* and basophilic differentiation in leukemia blasts remains to be elucidated.

Due to the paucity of ABL and AML with t(16;21), no guidelines for the treatment of this particular patient were available. Although leukemia was refractory to the idarubicin and cytarabine induction treatment, MEC salvage treatment led to remission at the molecular level. However, *FUS*‐*ERG* RT‐PCR briefly became positive, and after allogeneic hematopoietic stem cell transplantation, the disease showed florid relapse. Thus, it became apparent that currently available treatments for AML were unable to eradicate leukemia cells. FUS‐ERG was very recently shown to act as a transcriptional repressor of the retinoic acid signaling pathway and the treatment of t(16;21)‐AML cells with all‐*trans* retinoic acid led to reductions in the viability and differentiation of these cells 28. Thus, a clearer understanding of the molecular pathways affected by FUS‐ERG will facilitate the development of effective target therapy.

## Authorship

YT: main author, was responsible for writing and reviewing the manuscript, literature review, and provided direct care to the patient. YN: was responsible for writing and reviewing the manuscript. DS: performed electron microscopic analysis. CK: performed cytogenetic analysis. KT: performed cytomorphological analysis. KF: performed RT‐PCR and direct sequencing of *FUS‐ERG* fusion gene. MH: performed flow cytometric analysis. HO: was responsible for writing and reviewing the manuscript, literature review, and overall revision of the manuscript.

## Conflicts of Interest

The authors declare that there is no conflict of interest regarding the publication of this paper.

## References

[1] Arber, D. A. , L. Peterson , R. D. Brunning , J. Thiele , A. Crazi , M. M. Le Beau , et al. 2008 Acute myeloid leukaemia, not otherwise specified Pp. 130–139 *in* Swerdlow S. H., Campo E., Harris N. L., Jaffe E. S., Pileri S. A., Stein H., et al., eds. WHO classification of tumours of haematopoietic and lymphoid tissues. IARC, Lyon.

[2] Valent, P. , K. Sotlar , K. Blatt , K. Hartmann , A. Reiter , I. Sadovnik , et al. 2017 Proposed diagnostic criteria and classification of basophilic leukemias and related disorders. Leukemia 31:788–797. 28090091 10.1038/leu.2017.15 PMC7115817

[3] Mecucci, C. , A. Bosly , J. L. Michaux , A. Broeckaert‐Van Orshoven , and H. Van den Berghe . 1985 Acute nonlymphoblastic leukemia with bone marrow eosinophilia and structural anomaly of chromosome 16. Cancer Genet. Cytogenet. 17:359–363. 3860282 10.1016/0165-4608(85)90120-7

[4] Huret, J. L. 2005 t(16;21)(p11;q22). Atlas Genet. Cytogenet Oncol. Haematol. 9:36–38.

[5] Ichikawa, H. , K. Shimizu , Y. Hayashi , and M. Ohki . 1994 An RNA‐binding protein gene, TLS/FUS, is fused to ERG in human myeloid leukemia with t(16;21) chromosomal translocation. Cancer Res. 54:2865–2868. 8187069

[6] Panagopoulos, I. , P. Aman , T. Fioretos , M. Hoglund , B. Johansson , N. Mandahl , et al. 1994 Fusion of the FUS gene with ERG in acute myeloid leukemia with t(16;21)(p11;q22). Genes Chromosom. Cancer 11:256–262. 7533529 10.1002/gcc.2870110408

[7] Pereira, D. S. , C. Dorrell , C. Y. Ito , O. I. Gan , B. Murdoch , V. N. Rao , et al. 1998 Retroviral transduction of TLS‐ERG initiates a leukemogenic program in normal human hematopoietic cells. Proc. Natl. Acad. Sci. U. S. A. 95:8239–8244. 9653171 10.1073/pnas.95.14.8239 PMC20960

[8] Shvidel, L. , D. Shaft , B. Stark , M. Shtalrid , A. Berrebi , and P. Resnitzky . 2003 Acute basophilic leukaemia: eight unsuspected new cases diagnosed by electron microscopy. Br. J. Haematol. 120:774–781. 12614208 10.1046/j.1365-2141.2003.04167.x

[9] Kritharis, A. , J. Brody , P. Koduru , S. Teichberg , and S. L. Allen . 2011 Acute basophilic leukemia associated with loss of gene ETV6 and protean complications. J. Clin. Oncol. 29:e623–e626. 21576634 10.1200/JCO.2010.34.5710

[10] Duchayne, E. , C. Demur , H. Rubie , A. Robert , and N. Dastugue . 1999 Diagnosis of acute basophilic leukemia. Leuk. Lymphoma 32:269–278. 10037024 10.3109/10428199909167387

[11] ISCN . 2013 Structural Chromosome Rearrangements P. 140 *in* Schaffer L. G., McGowan‐Jordan J. and Schmid M., eds. An international system for human cytogenetic nomenclature. S. Karger, Basel.

[12] Kong, X. T. , K. Ida , H. Ichikawa , K. Shimizu , M. Ohki , N. Maseki , et al. 1997 Consistent detection of TLS/FUS‐ERG chimeric transcripts in acute myeloid leukemia with t(16;21)(p11;q22) and identification of a novel transcript. Blood 90:1192–1199. 9242552

[13] Dastugue, N. , E. Duchayne , E. Kuhlein , H. Rubie , C. Demur , J. Aurich , et al. 1997 Acute basophilic leukaemia and translocation t(X;6)(p11;q23). Br. J. Haematol. 98:170–176. 9233581 10.1046/j.1365-2141.1997.1562968.x

[14] Quelen, C. , E. Lippert , S. Struski , C. Demur , G. Soler , N. Prade , et al. 2011 Identification of a transforming MYB‐GATA1 fusion gene in acute basophilic leukemia: a new entity in male infants. Blood 117:5719–5722. 21474671 10.1182/blood-2011-01-333013

[15] Ducassou, S. , V. Prouzet‐Mauleon , M. C. Deau , P. Brunet de la Grange , B. Cardinaud , H. Soueidan , et al. 2017 *MYB*‐*GATA1* fusion promotes basophilic leukaemia: involvement of interleukin‐33 and nerve growth factor receptors. J. Pathol. 242:347–357. 28418072 10.1002/path.4908

[16] Peterson, L. C. , J. L. Parkin , D. C. Arthur , and R. D. Brunning . 1991 Acute basophilic leukemia. A clinical, morphologic, and cytogenetic study of eight cases. Am. J. Clin. Pathol. 96:160–170. 1862771 10.1093/ajcp/96.2.160

[17] Beer, P. A. , D. J. Knapp , P. H. Miller , N. Kannan , I. Sloma , K. Heel , et al. 2015 Disruption of IKAROS activity in primitive chronic‐phase CML cells mimics myeloid disease progression. Blood 125:504–515. 25370416 10.1182/blood-2014-06-581173 PMC4300391

[18] Seth, T. , A. Vora , M. Bhutani , K. Ganessan , P. Jain , and V. Kochupillai . 2004 Acute basophilic leukemia with t(8;21). Leuk. Lymphoma 45:605–608. 15160925 10.1080/10428190310001598053

[19] Lorsbach, R. B. , R. McNall , and S. Mathew . 2001 Marked bone marrow basophilia in a child with acute myeloid leukemia with a cryptic t(8;21)(q22;q22) chromosomal translocation. Leukemia 15:1799–1801. 11681427 10.1038/sj.leu.2402263

[20] Arber, D. A. , J. W. Vardiman , R. D. Brunning , L. Peterson , M. M. Le Beau , J. Thiele , et al. 2008 Acute myeloid leukaemia with recurrent genetic abnormalities Pp. 110–123 in Swerdlow S. H., Campo E., Harris N. L., Jaffe E. S., Pileri S. A., Stein H., et al., eds. WHO classification of tumours of haematopoietic and lymphoid tissues. IARC, Lyon.

[21] Pidala, J. , J. Pinilla‐Ibarz , and H. D. Cualing . 2008 A case of acute basophilic leukemia arising from chronic myelogenous leukemia with development of t(7;8)(q32;q13). Cancer Genet. Cytogenet. 182:46–49. 18328951 10.1016/j.cancergencyto.2007.12.009

[22] Giagounidis, A. A. , B. Hildebrandt , M. Heinsch , U. Germing , M. Aivado , and C. Aul . 2001 Acute basophilic leukemia. Eur. J. Haematol. 67:72–76. 11722593 10.1034/j.1600-0609.2001.t01-1-00487.x

[23] Kim, J. , T. S. Park , J. Song , K. A. Lee , D. J. Hong , Y. H. Min , et al. 2009 Detection of FUS‐ERG chimeric transcript in two cases of acute myeloid leukemia with t(16;21)(p11.2;q22) with unusual characteristics. Cancer Genet. Cytogenet. 194:111–118. 19781443 10.1016/j.cancergencyto.2009.06.010

[24] Morgan, R. , C. B. Riske , A. Meloni , C. A. Ries , C. H. Johnson , R. S. Lemons , et al. 1991 t(16;21)(p11.2;q22): a recurrent primary rearrangement in ANLL. Cancer Genet. Cytogenet. 53:83–90. 2036642 10.1016/0165-4608(91)90117-d

[25] Fukushima, Y. , N. Fujii , Y. Tabata , Y. Nishimura , T. Fusaoka , T. Yoshihara , et al. 2001 AML(M7) associated with t(16;21)(p11;q22) showing relapse after unrelated bone marrow transplantation and disappearance of TLS/FUS‐ERG mRNA. Rinsho. Ketsueki. 42:502–506. 11505530

[26] Sadamori, N. , E. Yao , M. Tagawa , H. Nakamura , I. Sasagawa , T. Itoyama , et al. 1990 16;21 translocation in acute nonlymphocytic leukemia with abnormal eosinophils: a unique subtype. Acta Haematol. 84:212–216. 2125791 10.1159/000205069

[27] Jekarl, D. W. , M. Kim , J. Lim , Y. Kim , K. Han , A. W. Lee , et al. 2010 CD56 antigen expression and hemophagocytosis of leukemic cells in acute myeloid leukemia with t(16;21)(p11;q22). Int. J. Hematol. 92:306–313. 20694842 10.1007/s12185-010-0650-5

[28] Sotoca, A. M. , K. H. Prange , B. Reijnders , A. Mandoli , L. N. Nguyen , H. G. Stunnenberg , et al. 2016 The oncofusion protein FUS‐ERG targets key hematopoietic regulators and modulates the all‐trans retinoic acid signaling pathway in t(16;21) acute myeloid leukemia. Oncogene 35:1965–1976. 26148230 10.1038/onc.2015.261 PMC4833872

